# Cortical time-course of evidence accumulation during semantic processing

**DOI:** 10.1038/s42003-023-05611-6

**Published:** 2023-12-08

**Authors:** Gayane Ghazaryan, Marijn van Vliet, Lotta Lammi, Tiina Lindh-Knuutila, Sasa Kivisaari, Annika Hultén, Riitta Salmelin

**Affiliations:** 1 Department of Neuroscience and Biomedical Engineering, Aalto University, P.O. Box 12200, FI-00076 Aalto, Finland; 2 Aalto NeuroImaging, Aalto University, P.O. Box 12200, Aalto, FI-00076 Finland

**Keywords:** Language, Neural decoding

## Abstract

Our understanding of the surrounding world and communication with other people are tied to mental representations of concepts. In order for the brain to recognize an object, it must determine which concept to access based on information available from sensory inputs. In this study, we combine magnetoencephalography and machine learning to investigate how concepts are represented and accessed in the brain over time. Using brain responses from a silent picture naming task, we track the dynamics of visual and semantic information processing, and show that the brain gradually accumulates information on different levels before eventually reaching a plateau. The timing of this plateau point varies across individuals and feature models, indicating notable temporal variation in visual object recognition and semantic processing.

## Abstract (web summary)

An MEG study on semantic processing of concepts in a silent picture naming task indicates that the brain gradually accumulates information before reaching a plateau.

## Introduction

Concepts are fundamental building blocks of our understanding of the world and communication with others. Brain regions associated with semantic knowledge have been extensively studied^1–4^, yet the mechanisms by which concepts are accessed in the brain are still not well understood. Object recognition is a common task that requires accessing concepts, and it is a prime target for experimental research on semantic processing. Despite how rapidly we can recognize an object, the process likely consists of multiple phases^5^, involving the interplay between visual and semantic properties^6^ and the emergence and accumulation of information over time^7,8^. In this work, we track and examine the dynamic progression of semantic processing in the human brain over the course of visual object recognition. In particular, we examine the accumulation of information through time.

Object recognition is essential in everyday functioning and, indeed, it has received great interest in human neuroscience^6,9–11^. The underlying process is thought to progress from a focus on low-level visual features to a focus on complex semantic representations^6^. Semantic knowledge models^12–15^ together with machine learning methods^16,17^ have been utilized to link brain activation patterns and semantic processing. Applying such methods to time-sensitive neuroimaging data, semantic processing has been shown to follow a coarse-to-fine progression^6^. Coarse semantic categories, but not individual concepts, can be discriminated based on earlier brain response patterns; by around 150 ms, it is possible to decode categories of objects^6^. Individual concepts, however, can be decoded only at later time points, around 300–450 ms after stimulus onset^7,18–20^.

Previous studies, which have focused on decoding at isolated time windows in sequence, have revealed a pattern of increasing decoding accuracy up to a peak, followed by a gradual decrease in accuracy^7,18,19^. Furthermore, cross-temporal decoding (decoding information from one time point with models trained on other time points) has shown that the underlying brain activation patterns evolve rapidly following stimulus onset, with some generalization of similar encoded information across nearby time points^21,22^. Such generalization indicates that information is maintained or accumulated from overlapping processes^8,23^.

**Fig. 1:**
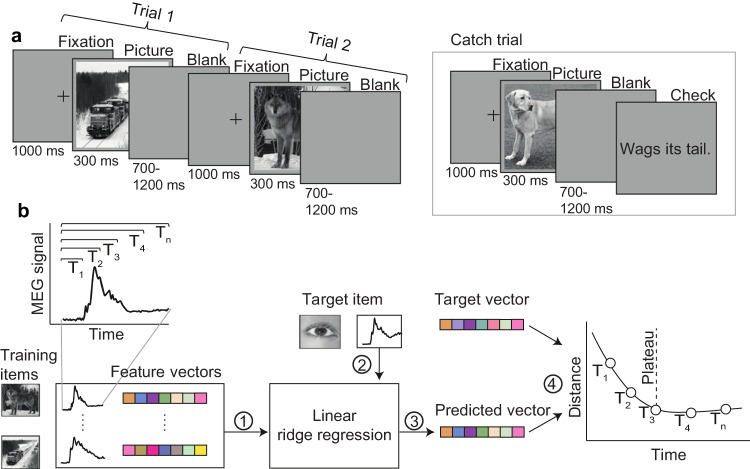
Overview of experiment and methodology. **a** Experimental paradigm. In each trial, participants were presented with a picture of an object and asked to identify it; randomly occurring catch trials ensured compliance. **b** Overview of the brain-to-semantics mapping method. Linear regression models were (1) trained on a set of brain response-semantic vector pairs and (2) tested on the brain response to one left-out concept, (3) yielding a predicted vector. (4) The distance between the predicted vector and the target vector was calculated. This procedure was repeated for all concepts. The same procedure was performed for each instance of the cumulative window. The dashed line corresponds to the point after which prediction-target distance no longer decreased (plateau point). Semantic vectors were created using the word2vec algorithm on a large Finnish text corpus.

To investigate how the brain processes information and accesses a concept, we used MEG (magnetoencephalography) brain response data from a picture viewing experiment, in which participants were shown pictures of objects and asked to silently identify them (Fig. 1a). This task focuses on object perception through to concept access^5^, and excludes later processes involving phonological forms and speech production. We contrast two approaches for decoding semantic representations: the traditionally used sliding approach taking one time point at a time, and a cumulative modeling approach (Fig. 1b) that widens the window at each time step. We demonstrate that, indeed, the brain gradually accumulates semantic information with eventual stabilization of accumulated information and access to a fully enriched object identity. For brain-level object recognition, it seems essential to take into account all information gathered up to a certain time point, instead of limiting the view to a sequence of single snapshots, analyzed in isolation.

## Results

Participants performed a silent visual concept identification task while MEG responses were recorded (Fig. 1a, see “Methods” for further details). We represented each concept as a semantic feature vector derived from a large text corpus using word2vec^24,25^, and trained models to predict these feature vectors from the MEG responses. To emphasize semantic properties of concepts over low-level visual features of specific stimulus images, we analyzed average brain responses to multiple different exemplars of the same concept. We performed analyses on both the grand average MEG responses, and separately for each participant. The MEG responses, spanning a period of 1000 ms after the stimulus onset, were downsampled and binned into 20 ms time points, resulting in time series of 50 points for each of the 60 concepts. Each time point contained MEG responses from 204 sensors. Following a neural decoding approach, we employed multivariate linear ridge regression to predict feature vectors from MEG responses (see “Methods” for further details).

In order to discriminate between conceptual and perceptual processes (known to be intertwined^26^), we compared the semantic feature model (word2vec) to a visual feature model that aims to describe visual object recognition in a manner that is similar to the primate visual cortex (CORnet)^27^. Visual feature vectors were derived by inputting the same graphical images that were shown to participants into the CORnet-S model that consists of four layers, corresponding to the cortical regions V1, V2, V4, and IT. Thus, we considered five different feature vector models: the word2vec model derived from a large text corpus; and four levels of the CORnet-S visual processing model. To evaluate predictive performance, we used a leave-one-concept-out zero-shot approach, in which trained models were tested on concepts that were excluded from the training sets. We used the prediction-target distance as a metric and investigated how this varied as a function of time.

### Grand Average

**Fig. 2:**
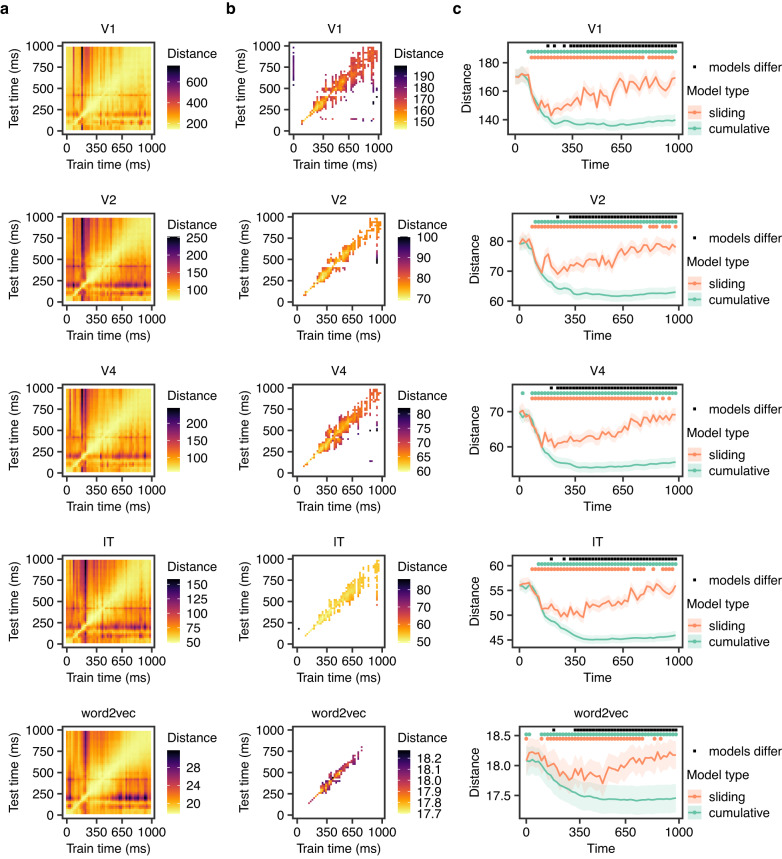
Results of grand average analysis. **a** Cross-temporal decoding results on grand average data. Here, models were trained on one 20-ms time point and tested on another 20-ms time point. The color corresponds to the prediction-target distance averaged over all targets. **b** Temporal cross-decoding results on grand average data. Only statistically significant values shown (*p* < 0.05, FDR corrected, based on permutation tests with 1000 permutations). **c** Distance between predictions and targets over time on grand average data, using two different types of models: a sliding model taking one 20-ms time point at a time, without overlap, and a cumulative window with width increasing at 20-ms increments. The lines represent the mean across concepts, and the edges of the shaded areas indicate plus and minus one standard error. The dots above each line plot indicate time points with statistically significant differences (*p* < 0.05), based on permutation tests with 1000 permutations, FDR corrected). Note that due to differences in the feature space of the models, the magnitudes of the Euclidean distance values are on different scales, and should not be directly compared. Instead, the temporal patterns are the focus of interest. For further details on the calculation of distance, see “Methods”.

**Fig. 3:**
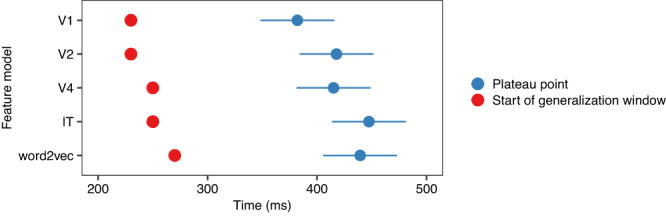
Comparison of generalization window and plateau points. Blue dots indicate estimated marginal means (95% confidence intervals) from a linear mixed model predicting plateau points from the feature model with random by-concept intercepts. Red dots show the start of the generalization window, identified from the cross-temporal decoding, for comparison.

Using grand average data, we first sought to examine the degree of shared encoded information between different time points. To do this, we employed a cross-temporal decoding approach in which models are trained on data from one time point and tested on another time point. All feature models indicated significant generalization of information encoded in the brain response (*p* < 0.05, based on a permutation test with 1000 permutations, FDR corrected) (Fig. 2a, b). The generalization was most pronounced for consecutive time points, with further away time points showing less generalization. The start of this window, defined as the point after which there was significant generalization between consecutive time points in the cross-temporal decoding (Fig. 2b), varied from 250 (V1, V2) to 270 ms (word2vec) (Fig. 3).

We examined the underlying reason for this generalization by considering two alternatives: either the encoded information is maintained, or accumulation of information continues throughout this period. For each feature model, we compared two different types of models with (1) sliding and (2) cumulative approaches. All sliding models showed a decrease in prediction-target distance over time until a trough, followed by an increase in distance. In contrast, the cumulative models showed decreasing prediction-target distance, with an eventual plateau (Fig. 2c). Both approaches yielded mean prediction-target distances significantly lower than chance at some time points (*p* < 0.05, based on a permutation test with 1000 permutations, FDR corrected). For the sliding models, time points of significantly lower than chance distance were between 140 to 760 ms for word2vec, 100 ms to 780 ms for V1, 80 to 760 ms for V2, 80 to 780 ms for V4, and 80 to 680 ms for IT. For the cumulative models, corresponding time points were 100 ms onwards for word2vec, 100 ms onwards for V1, 80 ms onwards for V2, 80 ms onwards for V4,  and 120 ms onwards for IT. The cumulative model eventually yielded significantly lower distance compared to sliding models for all feature models (*p* < 0.05, based on a permutation test with 1000 permutations) from 320 ms onwards for word2vec, and from 340 ms/320 ms/240 ms/320 ms onwards for the visual feature models V1/V2/V4/IT. Results are shown in Fig. 2c.

As the cumulative model performed better than the sliding model, the next target of interest was the time when the cumulative model plateaued, as this would indicate whether or not information was accumulated during the generalization window. We reasoned that if the cumulative models plateaued close to the same time as the generalization began, this would indicate that little new information was encoded in the patterns during the generalization window, and the generalization was purely due to maintenance. Alternatively, if the plateau occurred substantially later, this would indicate that new information was encoded in the brain signal during this period, and there was information accumulation.

We defined plateau points as the time point at which the prediction-target distance no longer meaningfully decreased. We opted for a threshold of 5% for this, meaning that when the model had reached within 5% of the total reduction in distance, we classified it as reaching plateau. We chose a 5% threshold rather than the global minimum whose exact time point could be rather arbitrary due to noise-induced signal variation. We interpreted this plateau point as the time after which little more relevant information was encoded in the MEG signal. We then used a mixed effects linear regression to predict plateau points from the feature model used, with random intercepts for each concept.

Figure 3 shows estimated plateau points in comparison to the points when the generalization window began. The plateaus were significantly later than the start of the generalization window for all models, *p* < 0.05. This indicates that further relevant information is encoded in the MEG signal during this period. In other words, there is information accumulation rather than only maintenance.

The estimated mean (95% CI) plateau was 382 (348–416) ms for V1, 418 (384–451) ms for V2, 415 (381–449) ms for V4, 447 (414–481) ms for IT and 439 (405–473) ms for word2vec. Apart from V2 and V4 (*p* = 0.9803), and IT and word2vec (*p* = 0.4406), the estimates were significantly different (all *p* < 0.0001, Tukey correction).

### Individual level results

**Fig. 4:**
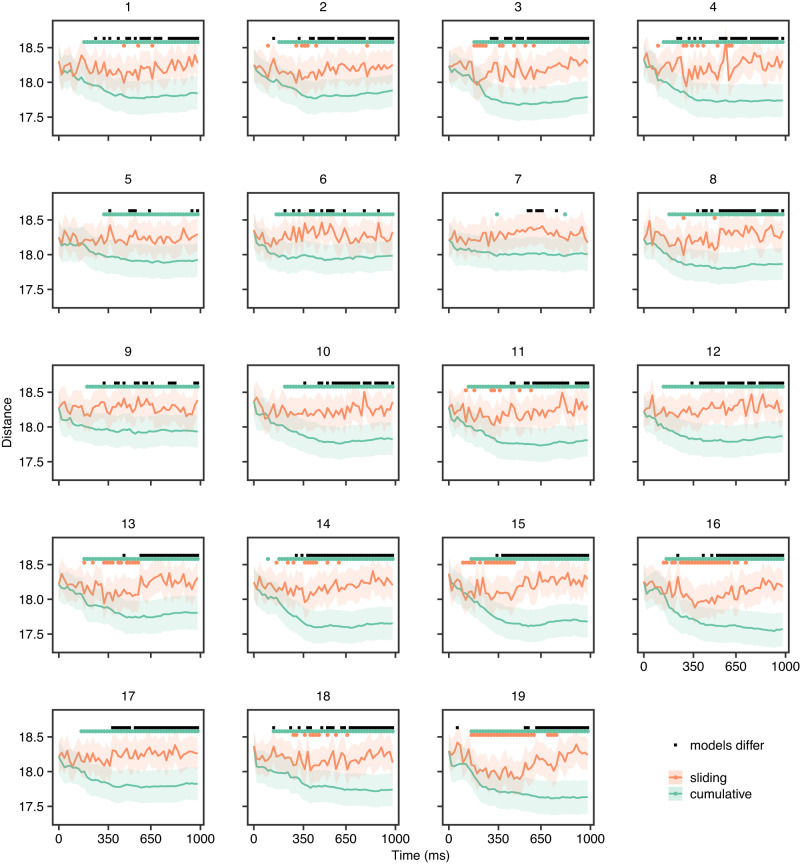
Distance between predictions and targets over time in individual participants for semantic model. The lines represent the mean across concepts, and the edges of the shaded areas indicate plus and minus one standard error. The dots above each line plot indicate time points with statistically significant differences (*p* < 0.05), based on permutation tests with 1000 permutations, FDR corrected). All participants displayed decreasing prediction-target distance as a function of time with an eventual plateau. Plateau points (for cumulative models) and troughs (for sliding fixed-length models) were defined as the time point after which prediction-target distance no longer decreased by more than 5%.

Following the grand average analyses, we investigated whether a consistent pattern could be identified at the individual level and explored individual differences therein. We again compared the sliding and cumulative models to investigate the progression of information processing of each participant. The results for word2vec are presented in Fig. 4 and the visual feature models are presented in Supplementary Figs. 1–4. All participants displayed decreasing prediction-target distance as a function of time with an eventual plateau for cumulative models, which yielded significantly better than chance predictions (based on a permutation test with FDR correction, *p* < 0.05). The sliding models in word2vec produced significantly better than chance predictions at some time points (based on a permutation test with FDR correction, *p* < 0.05) for the majority of participants (but not 5, 6, 7, 10, 12, 17).

**Fig. 5:**
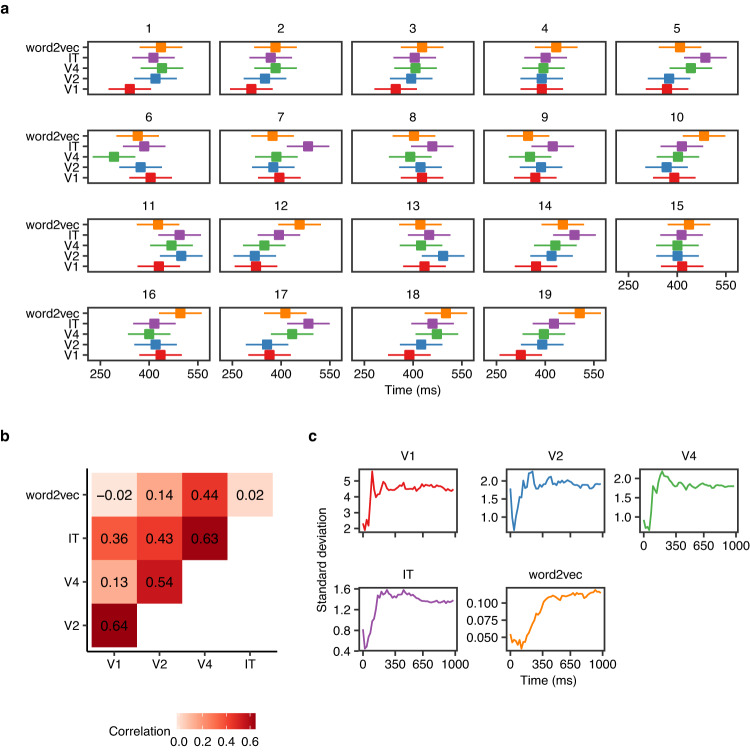
Variation of plateau points and prediction-target distance between participants. **a** Estimated plateau means (95% confidence intervals) for each participant based on linear mixed model. **b** Correlation between estimated plateau points of the different feature models. **c** Standard deviation between participants of prediction-target distance (mean over concepts) as a function of time, for each feature model.

A linear mixed model (with random intercepts for each concept) showed significant variation in plateau points between participants and feature models (Fig. 5a, Table 1). There was a significant main effect of the feature model (*F*(4, 5546) = 3.88, *p* = 0.001) following a similar pattern observed in the grand average analyses with IT and word2vec plateauing significantly later than V1 and V2 (*p* < 0.05), see Table 2. We also observed a significant main effect of participants indicating inter-individual variability (*F*(18, 5546) = 9.23, *p* = 0.001).

**Table 1:** Mixed-model regression analysis of plateau times.

Effect	df	*F*	*p*-value
Participant	18, 5546	3.88	<0.001
Feature model	4, 5546	9.23	<0.001
Participant × Feature model	72, 5546	1.13	0.271

**Table 2:** Estimated plateau points for each feature model.

Feature model	Min estimate	Max estimate	SE
V1	308 ms	435 ms	33 ms
V2	319 ms	499 ms	33 ms
V4	292 ms	473 ms	33 ms
IT	368 ms	494 ms	33 ms
word2vec	347 ms	506 ms	33 ms

We explored how plateau timings in one feature model were related to those in other feature models. The visual feature models were positively correlated and higher correlations were observed between consecutive layers such that participants with earlier plateaus in V1 had earlier plateaus in V2; similarly for V2–V4 and V4–IT (Fig. 5b and Supplementary Figure 5). We also examined how the differences between participants in prediction-target distance changed as a function of time. Specifically, we looked at the time points when differences between participants reached their maximum. The model based on word2vec appeared to reach maximum variation across participants later compared to visual feature models (Fig. 5c).

### Representational similarity analysis

**Fig. 6:**
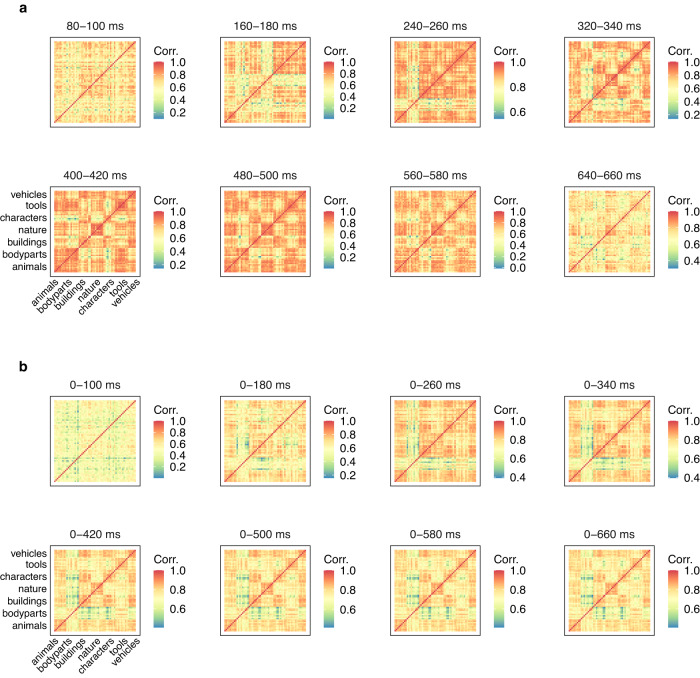
Concept-to-concept brain signal dissimilarity matrices (sensor-level) plotted over time, averaged across all participants. **a** Sliding approach. **b** Cumulative approach.

**Fig. 7:**
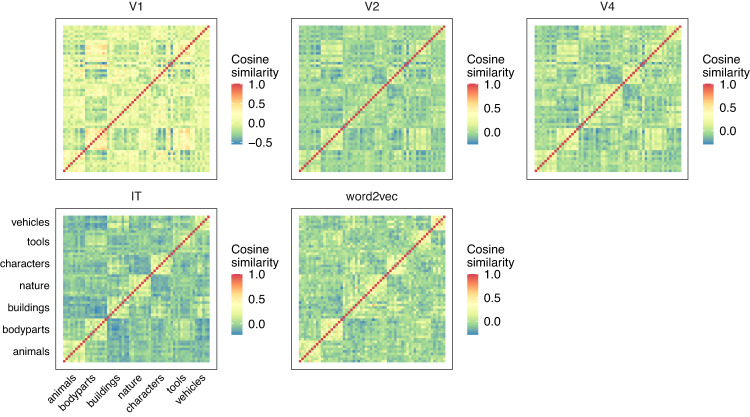
Concept-to-concept similarity for different feature models. Pairwise cosine similarity between feature vectors for each concept is shown and categorical structure can be seen along the diagonals.

**Fig. 8:**
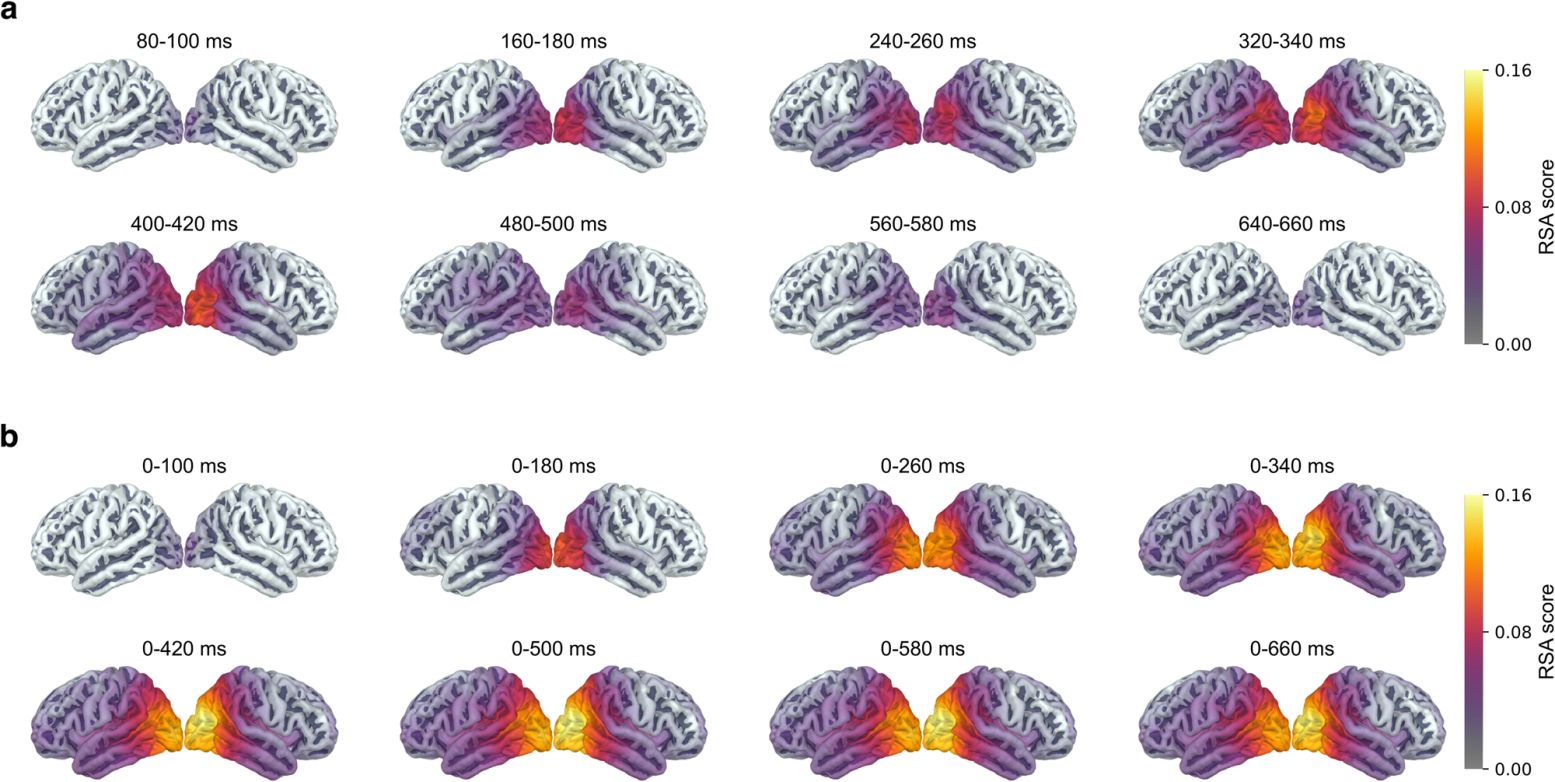
RSA maps for the semantic feature model. RSA maps illustrating the statistically significant clusters for different time windows, with **a** the sliding fixed-length approach and **b** the cumulative approach. For details on calculations of RSA scores see Methods. We used a cluster permutation test^61^ across participants with a cluster threshold of *p* = 0.01, a cluster-wide significance threshold of *p* = 0.05 and 5000 permutations in accordance with Hultén et al.^38^.

To investigate the brain areas involved in the information accumulation processes, we performed representational similarity analysis (RSA) between concept similarity in the brain (the brain-level concept-to-concept dissimilarity matrices at different time points in sliding and cumulative approaches; Fig. 6a, b) and concept similarity in the feature models (the feature model concept-to-concept dissimilarity matrices; Fig. 7). We observed the highest RSA scores in the occipital regions of both hemispheres (Fig. 8a, b). Incrementally adding time points did not change the regions where the RSA scores were highest. The 0–420 ms and onward windows showed the highest correlations. This timing aligned with the decoding results described above. RSA figures for the visual feature models are presented in Supplementary Figs. 6–9.

## Discussion

In this study, we tracked the progression of information processing throughout visual object recognition. We identified a period of information generalization, and found that during this time there is information accumulation, during which concept representations are enriched. This interpretation is in line with Contini et al.^8^, who suggested two reasons for information generalization: (1) the encoded representation is maintained throughout this period, or (2) there are ongoing overlapping processes of differing duration, such that some information is maintained while the representation is enriched with further accumulation. By demonstrating information accumulation in this window, our work brings relevant new findings to complement earlier studies on object recognition^28,29^ and semantic access in general^7,30–37^, especially regarding its temporal progression^7,18,19,38^.

We demonstrated information accumulation with the help of models that mapped between MEG responses and concepts, and showed that these models generalize to new concepts (following a zero-shot approach^17^). We compared a semantic feature model (word2vec) to models using visual features generated from CORnet, a model mimicking visual processing in primate visual cortex (V1, V2, V4, and IT). Through this comparison, and by using static images and multiple instances of each concept, we aimed to reduce the effects of mere stimulus characteristics on the observations and highlight neural effects related to semantic processing of the concept. Importantly, semantic features were derived from a large text corpus, and not directly from the limited set of visual stimuli shown to the participants. The visual feature representations were derived by applying the pre-trained CORnet visual model to the stimuli the participants were shown. When comparing visual and semantic models it is important to recognize that such models are always simplifications of the true underlying processes. In this particular case, the visual feature models may not capture all underlying visual processes. As such, differences between the visual models and the semantic model may not only indicate semantic processing, but rather inadequacies in the visual models^26^. Future work may avoid the confounding between visual and semantic processing by focusing on written words^38–40^, however, written words are known to be a more challenging medium for neural decoding^41^.

The sliding approach indicated that there was information relevant to concepts starting at about 80–100 ms, which matches the timing reported in previous work^7,18,26,42,43^. Cross-temporal decoding indicated that there was also generalization in the brain signal from about 250 ms onwards. The cumulative models resulted in significantly lower prediction-target distance than the sliding models in all feature models, from 240–340 ms onwards, indicating there is information accumulation.

Models based on lower-level visual features (V1, V2) plateaued earlier than the semantic feature model (word2vec) or high-level visual feature models (IT). This pattern matches the level of processing. Furthermore, we found that plateau points of consecutive layers in the visual feature model were systematically correlated. Although word2vec showed moderate correlation with V4, there was indication that individual variability in word2vec decoding was delayed compared to the visual models. This suggests that the semantic feature model is capturing information other than visual feature correlates, and the decoding is not just relying on features correlated with low-level visual processing.

We propose that the plateau point of the semantic model could be interpreted as the time point after which the representation of the concept is no longer enriched. On group-level data, the mean plateau time across concepts was around 450 ms. This, coupled with the generalization shown between 270 and 750 ms, indicates that there is likely both accumulation of information (preceding plateau) and maintenance (following plateau). The timing of the plateau point varied between participants, with means ranging from approximately 350 to 500 ms.

Bo et al.^44^ showed that visual activity appears in different regions up to around 360 ms. Peaks in conceptual processing (when controlling for visual features) have been shown to occur at different points between 180 and 540 ms^26^. The plateau points observed here, while not directly comparable due to differing methodologies, line up with these previous results.

Disentangling accumulation from maintenance, however, may not be as simple in the presence of noise. In a situation where there is only maintenance, but the recorded signals have substantial noise, a similar pattern of decreasing prediction-target distance might be observed, as adding more time points would counteract the noise and improve performance. Based on the behavior of the cross-temporal, cumulative, and sliding models, however, we consider this to be a less likely explanation than accumulation. Specifically, we refer to the following observations: First, the cross-temporal models indicated that while there was some generalization, this was predominantly between consecutive time points. If there was a constant signal with noise, we would not expect such a difference between consecutive and non-consecutive cross-temporal decoding performance. Second, the sliding models showed an increase and then decrease in predictive performance, rather than a sustained level of performance, counter to what would be expected in pure maintenance. Third, significant prediction by the sliding models continued well past the plateau of the cumulative models. If the cumulative models were simply accommodating for noise in the data, we could expect the increase in performance to continue until the signal was no longer predictive.

RSA indicated that occipital areas were relevant to semantic processing of pictures and showed temporal patterns in accordance with the decoding approach, with higher RSA scores at time points when decoding performed better. Interestingly, brain regions that are consistently reported in studies of picture naming^45^, such as the left temporal and left parietal cortices, did not strongly account for semantic relationships between target concepts. However, our results align with Simanova et al.^43^, who suggested that the predominance of occipital areas may be due to inherent visual similarities between semantically similar objects. In other words, the appearance of an object is tied to its semantic meaning, so it is unsurprising that brain regions related to visual processing emerged in the RSA analysis. While it is possible that silent identification of the pictures did not activate the phonological form of the concept as strongly as an overt naming task would have done, the lack of involvement of the typical language areas may be a reflection of the fact that semantic similarity is not mirrored in phonological similarity (for example ‘cat’ and ‘dog’ are semantically near but phonologically distant).

The fact that people agree on names of objects and can communicate about them indicates that there are commonalities in semantic understanding. However, as each person has a unique life experience, the underlying semantic processes are also likely to vary. Previous studies have reported inter-individual variation in behavioral measures of naming speed^46^, neural correlates of semantic representation^47^, and gaze-behavior measures of visual salience^48^. Individual variation has also been indirectly investigated through cross-decoding between individuals. This is performed by training models on data from one or more individuals and testing on data from another individual^32,33,49^. Generally, such cross-decoding has been less accurate than within-individual decoding. As these studies predominantly used imaging methods that favor high spatial precision over temporal precision, the results likely indicate individual variation in the cortical areas involved in language processing, the existence of which has been known since early studies^50^. Individual differences in the temporal domain of semantic processing have also recently been indicated by Rupp et al.^19^, who reported individual variation in the windows when decoding performed best, suggesting differences in the progression of semantic understanding. Here, we found individual variability in accumulation of visual and semantic information. This finding is relevant to, for example, development of brain-computer interfaces, where individual variability may need to be taken into account. Variability between concepts is also an intriguing question for future studies but will likely require more repetitions of each concept than in the present study to ensure less noisy cortical time courses for individual concepts.

We have presented here a new perspective on the temporal dynamics of semantic understanding that opens future avenues of research. These include a deeper understanding of individual cognitive variation, addressing the link to behavioral measures, adapting to other modalities such as spoken or written words, and investigating concept processing in context by using more naturalistic stimuli such as sentences or stories. Such research will bring us towards a more complete model of language within the brain.

## Methods

### Participants

Twenty native speakers of Finnish (females/males 10/10, age range 20–27, mean age 22) participated in the study. All participants were right handed (Edinburgh handedness questionnaire^51^) and had normal or corrected to normal vision. The study was approved by the Aalto University Research Ethics Committee and participants provided written informed consent prior to their participation. All ethical regulations relevant to human research participants were followed. Data from 1 participant was excluded due to technical issues with the MEG recordings, leaving data from 19 participants in the final analysis.

### Stimuli and procedure

Stimuli consisted of 300 grayscale photographic images of 60 concrete Finnish nouns (five different depictions of each). To minimize the effects of low-level visual features on the neural responses, there were five different images depicting each concept. Overall, each concept was presented in picture form 18 times (across three sessions on different days), the responses of which were averaged.

The concepts belonged to 7 different categories: animals, body parts, buildings, nature, human characters, tools/artifacts, and vehicles. Details of the nouns are presented in Supplementary Table 1. There were nine concepts in each category except for vehicles, which had six concepts. In the experiment, the concepts were also presented in written and auditory forms in separate trials; the responses from those trials were not analyzed in this study.

Participants were tasked with viewing each picture and silently identifying and thinking about the depicted object. The stimuli were presented at a size of 106 × 106 mm on a screen 140 cm from the participants’ eyes, corresponding to a visual angle of 4.3^∘^. Each trial started with a fixation cross displayed for 1000 ms. The picture was then shown for 300 ms, followed by a blank screen for a randomized duration of 700–1200 ms (Fig. 1a).

To ensure that participants remained engaged during the experiment, we included comprehension tasks after 10% of trials. In these tasks, participants used optical response pads to indicate whether or not a written description was characteristic of the previously shown concept. As these tasks occurred after the trials, they did not interfere with the responses, thus all trials were included in the analysis.

### Data acquisition

MEG measurements were conducted at the Aalto NeuroImaging MEG Core (Aalto University, Espoo, Finland) using a Vectorview whole-head MEG system (MEGIN (Elekta Oy), Helsinki, Finland). The system has 306 sensors (204 planar gradiometers, 102 magnetometers). The head position was continuously tracked during the experiment by 5 head position indicator (HPI) coils placed at known locations with respect to identifiable anatomical landmarks. Eye movements and blinks were captured using 2 electrode pairs (one pair positioned above and below the left eye, the other in the corner of each eye). The recording was bandpass-filtered at 0.03–330 Hz and sampled at 1000 Hz. Anatomical MRIs were obtained using Siemens Magnetom Skyra 3.0 T MRI scanner with a T1-weighted MP-RAGE sequence at the Aalto NeuroImaging Advanced Magnetic Imaging (AMI) Centre.

### Data preprocessing

MEG data was first visually inspected and noisy channels were identified. External sources of noise were then removed using spatiotemporal signal space separation (tSSS)^52^ with Elekta Maxfilter software (MEGIN Oy, Finland). For each participant, data from different sessions was transformed to the same head position. All subsequent analysis was performed using the MNE-Python software package^53^. The data was low-pass filtered at 40 Hz and split into 1200 ms epochs, the first 200 ms of which was the pre-stimulus baseline interval. To reduce contamination related to heartbeats, eye movements, and blinks, we performed independent component analysis (ICA). In order to minimize the effect of slow drifts on ICA decomposition, we used continuous data high-pass filtered at 1 Hz^54^. Components corresponding to heartbeats, eye movements, and blinks were visually identified and excluded from epochs. Epochs corresponding to the same concept were then averaged, the time period between 0 and 1000 ms was extracted, and the signal was downsampled to create 20 ms bins. Only data from the gradiometers was used in the final analysis. This resulted in a matrix with 60 concepts × 204 channels × 50 time points for each participant.

We computed the source-level estimate of the average response to each concept using minimum norm estimates (MNE)^53^. Anatomical MRIs were used to reconstruct the cortical surface of each participant applying the FreeSurfer software package^55–57^. We used a single-layer boundary element model (BEM) with an icosahedron mesh of 2562 vertices in each hemisphere. When computing the inverse solution, a loose orientation constraint of 0.3 and depth weighting parameter of 0.8 were used. An empirical noise-covariance matrix was computed based on the pre-stimulus 200 ms interval to all concepts. To prepare the data for group-level analysis, participant-level source estimates of each concept were morphed to the FreeSurfer standard template brain (fsaverage).

### Semantic features

Semantic vector representations for the stimuli were obtained using the word2vec tool with skip-gram architecture and negative sampling algorithm^25^. Each concept was represented as a vector of length 300 which defines its location in semantic space. The components of a vector are based on word co-occurrence statistics in large text corpora, the Finnish Internet Parsebank^24^, which is based on a large sample (1.5 billion words) of Finnish language websites. Co-occurrence was considered to take place when a word appeared within a window from 5 words before to 5 words after the word corresponding to the concept of interest.

### Visual features

The CORnet model is a neural network architecture designed to simulate the processing of visual information in the primate brain. It consists of multiple layers of artificial neurons that are modeled after the neurons found in the primary visual cortex (V1), secondary visual cortex (V2), visual area V4, and the inferior temporal cortex (IT). V1, V2, V4, and IT form a hierarchy of visual processing, with each region responsible for processing increasingly complex visual information. Beginning with low-level features such as orientation and color, each region builds upon the previous one to construct a more complete representation of visual stimuli. This process culminates in high-level visual processing, such as object recognition^27^.

We created visual feature vectors by inputting the grayscale images into the CORnet-S model, and saving the outputs of each layer. We reduced the dimensionality of the visual feature vectors using PCA (principal components analysis). Following the zero-shot approach, we did this transformation in a cross-validated manner by first leaving out the exemplars of the left-out test concept, and then calculating the principal components on the training set. We then projected both the training and test vectors onto 295 principal components (the largest possible for the size of the training set) and averaged the transformed feature vectors across exemplars to arrive at one visual feature vector for each concept (per iteration of the cross-validation). For RSA, the procedure was the same but without leaving out concepts.

### Regression models

We used a zero-shot decoding approach^17^. Regularized multivariate ridge regression, as implemented in scikit-learn^58^, was used to fit a model that predicts the semantic feature vector of a target concept based on brain response. The sensor-level MEG responses were first standardized with respect to concepts, such that the mean of each predictor (time point-channel pair) was 0 and the standard deviation was 1. We used leave-one-out cross validation, such that models were trained on 59 out of the 60 concepts and evaluated on the remaining concept the model had not been trained on, for all permutations.

For the regression models, we compared prediction and target vectors using Euclidean distance. The Euclidean distance matches the loss function of linear regression. As the Euclidean distance to the training items is minimized during model fitting, it is appropriate to use this metric to assess the predictive performance on the test items. Note that due to differences in the feature space of the models, the magnitudes of the Euclidean distance values are on different scales and not directly comparable. Instead, the temporal patterns are the focus of interest.

### Mapping brain response to semantic space as a function of time

In accordance with Carlson et al.^21^ and Grootswagers et al.^22^, we performed cross-temporal decoding, in which models were trained and tested on different time windows to check whether there is information generalization in the brain across different time points. For this we trained and tested models on pairs of 20-ms time windows on data averaged over all participants. To explore the progression of semantic understanding in more detail we compared two types of models on average sensor-level MEG responses. First, we used sliding windows of fixed length similar to Sudre et al.^18^ and Rupp et al.^19^. Second, we developed a method to examine cumulatively widening windows (see details below). We evaluated models based on prediction-target distance (smaller distance indicates that the model better predicts the target concept). Both types of models were evaluated using leave-one-out cross validation.

For sliding fixed-length window models, regression models were trained and tested on fixed-length subsets of MEG data. A sliding window of 20 ms was used, with no overlap between adjacent windows. Each subset was evaluated independently of others. Similar models have been used by Sudre et al.^18^, Rupp et al.^19^, Hultén et al.^38^ and we expected a pattern of gradual decrease, followed by an increase in prediction–target distance.

For cumulative window models, regression models were trained and tested on cumulative subsets of MEG data. The window size was sequentially increased by 20 ms. Thus, all previously encoded information was included in the estimation and model evaluation (Fig. 1b).

### Identifying individual differences

To compare the progression of semantic understanding between participants, we used the cumulative models. We first calculated the progression of semantic information for all concepts for each participant. We then focused on the point of plateau, the time point after which there was less than 5% further decrease in prediction-target distance. We compared these plateau points using a linear mixed model, with random intercepts for concepts.

### Representational similarity analysis

We performed representational similarity analysis (RSA)^59^ on source localized MEG data to extract the brain areas accounting for highest similarity between the brain activation patterns and vector representations of the concepts for different time windows. This was done using the MNE-RSA software package^60^. RSA was performed for both sliding and cumulative time windows.

The model dissimilarity matrices (DSM) were obtained by calculating pairwise cosine distances between feature vectors. This was followed by the calculation of brain DSMs for each participant at each source-level vertex with a searchlight patch radius of 2 cm for the time window of interest. Brain DSMs were computed by calculating pairwise Pearson correlation coefficient between brain signals in response to different stimuli. The relationship between the brain DSMs and model DSMs was quantified by Spearman rank correlation coefficients for each participant. This resulted in participant-level RSA maps. We then used a cluster permutation test^61^ across participants with a cluster threshold of *p* = 0.01, a cluster-wide significance threshold of *p* = 0.05 and 5000 permutations in accordance with Hultén et al.^38^.

### Statistics and reproducibility

Statistical significance between regression model performance was evaluated using permutation tests with 1000 iterations as in Kivisaari et al.^30^. For each permutation, models were trained and evaluated. For the individual level analysis, this was done separately for each participant. *p*-values were calculated by the proportion of test statistics from the permuted data sets that were at least as high as the test statistics from the observed data.

When comparing prediction-target distance to chance-level, the mean distance over all concepts was used. When comparing the two types of models, a paired *t* statistic (across concepts) was calculated and compared to the permutation distribution for each time point. *p* values were corrected using false discovery rate (FDR) correction.

For RSA we used a cluster permutation test^61^ across participants with a cluster threshold of *p* = 0.01, a cluster-wide significance threshold of *p* = 0.05 and 5000 permutations in accordance with Hultén et al.^38^.

### Reporting summary

Further information on research design is available in the Nature Portfolio Reporting Summary linked to this article.

### Supplementary information

Supplementary Information

Reporting Summary

## Data Availability

The text corpus containing 1.5 billion Finnish words used to derive the statistical model cannot be publicly distributed due to the Finnish copyright law limitations. For further information see https://turkunlp.org/finnish_nlp.html#parsebank. The stimulus concepts are listed in the Supplementary Table 1. The MEG and MRI data are available upon reasonable request from the authors; the data is not publicly available as it contains personal information, and its reuse for other research purposes requires a new ethical pre-review. Numerical data for the figures that do not contain individual participant data is available at https://zenodo.org/doi/10.5281/zenodo.10076376^62^.

## Code availability

For preprocessing we used Elekta Maxfilter software (MEGIN Oy, Finland) and MNE-Python^53^. For the primary analysis we used Python and made substantial use of MNE-Python^53^, scikit-learn^58^, NumPy^63^, and Matplotlib^64^. In addition, we used R and the following packages: afex^65^, lme4^66^, qvalue^67^, ggplot2^68^. The feature vectors used in this study, together with the custom code used in the study can be accessed at https://zenodo.org/doi/10.5281/zenodo.10076376^62^. The code to compute RSA can be found at https://github.com/wmvanvliet/mne-rsa.
