# Supplementary material for: Cortical time-course of evidence accumulation during semantic processing
Source: Commun Biol. 2023 Dec 8;6:1242. doi: 10.1038/s42003-023-05611-6 (PMC10709650; doi:10.1038/s42003-023-05611-6)
Supplement: Supplementary file 1 — Supplementary Information [file 42003_2023_5611_MOESM1_ESM.pdf]

# Supplementary information for “Cortical time-course of evidence accumulation during semantic processing”

Gayane Ghazaryan[1*], Marijn van Vliet[1], Lotta Lammi[1], Tiina Lindh-Knuutila[1], Sasa Kivisaari[1], Annika Hultén[1,2], and Riitta Salmelin[1,2]

[1]Department of Neuroscience and Biomedical Engineering, Aalto University, P.O. Box 12200, FI-00076, Aalto, Finland
[2]Aalto NeuroImaging, Aalto University, P.O. Box 12200, Aalto, FI-00076, Finland
[*]Corresponding author gayane.ghazaryan@aalto.fi

**Supplementary Table 1: Linguistic properties of words corresponding to the visual stimuli.**

| Finnish word | English translation | Category | Letter count | Lemma freq. |
|---|---|---|---|---|
| koira | dog | animal | 5 | 1148512 |
| hevonen | horse | animal | 7 | 600026 |
| kissa | cat | animal | 5 | 410397 |
| hiiri | mouse | animal | 5 | 118922 |
| lammas | sheep | animal | 6 | 79046 |
| karhu | bear | animal | 5 | 78634 |
| leijona | lion | animal | 7 | 58120 |
| ankka | duck | animal | 5 | 20623 |
| kotka | eagle | animal | 5 | 14917 |
| käsi | hand | body part | 4 | 1881198 |
| silmä | eye | body part | 5 | 1221875 |
| jalka | foot | body part | 5 | 921001 |
| suu | mouth | body part | 3 | 520459 |
| selkä | back | body part | 5 | 422099 |
| korva | ear | body part | 5 | 341449 |
| sormi | finger | body part | 5 | 231641 |
| nenä | nose | body part | 4 | 148989 |
| varvas | toe | body part | 6 | 72551 |
| kirkko | church | building | 6 | 965363 |
| tie | road | building | 3 | 834283 |
| kirjasto | library | building | 8 | 375156 |
| museo | museum | building | 5 | 197654 |
| tehdas | factory | building | 6 | 174675 |
| linna | castle | building | 5 | 116508 |
| vankila | prison | building | 7 | 113426 |
| silta | bridge | building | 5 | 110498 |
| torni | tower | building | 5 | 68817 |
| lapsi | child | character | 5 | 3775414 |
| opettaja | teacher | character | 8 | 613284 |
| lääkäri | doctor | character | 7 | 582297 |
| poliisi | police | character | 7 | 517522 |
| kuningas | king | character | 8 | 254286 |
| pappi | priest | character | 5 | 205756 |
| tuomari | judge | character | 7 | 195403 |
| sotilas | soldier | character | 7 | 139619 |
| vanki | prisoner | character | 5 | 105450 |
| saari | island | nature | 5 | 294931 |
| meri | sea | nature | 4 | 293854 |
| puisto | park | nature | 6 | 156118 |
| joki | river | nature | 4 | 151140 |
| vuori | mountain | nature | 5 | 133552 |
| pilvi | cloud | nature | 5 | 117759 |
| pesä | nest | nature | 4 | 99988 |
| aalto | wave | nature | 5 | 77540 |
| kallio | rock | nature | 6 | 66610 |
| kirja | book | tool | 5 | 1687530 |
| pallo | ball | tool | 5 | 342738 |
| sormus | ring | tool | 6 | 46015 |
| saha | saw | tool | 4 | 35625 |
| lusikka | spoon | tool | 7 | 32322 |
| haarukka | fork | tool | 8 | 23395 |
| sakset | scissors | tool | 6 | 21218 |
| lapio | shovel | tool | 5 | 13855 |
| kampa | comb | tool | 5 | 12664 |
| auto | car | vehicle | 4 | 1773191 |
| juna | train | vehicle | 4 | 239963 |
| bussi | bus | vehicle | 5 | 220867 |
| laiva | ship | vehicle | 5 | 219676 |
| vene | boat | vehicle | 4 | 205989 |
| rekka | truck | vehicle | 5 | 30658 |

**Supplementary Fig. 1: Distance between predictions and targets over time in individual participants for V1 visual model.**

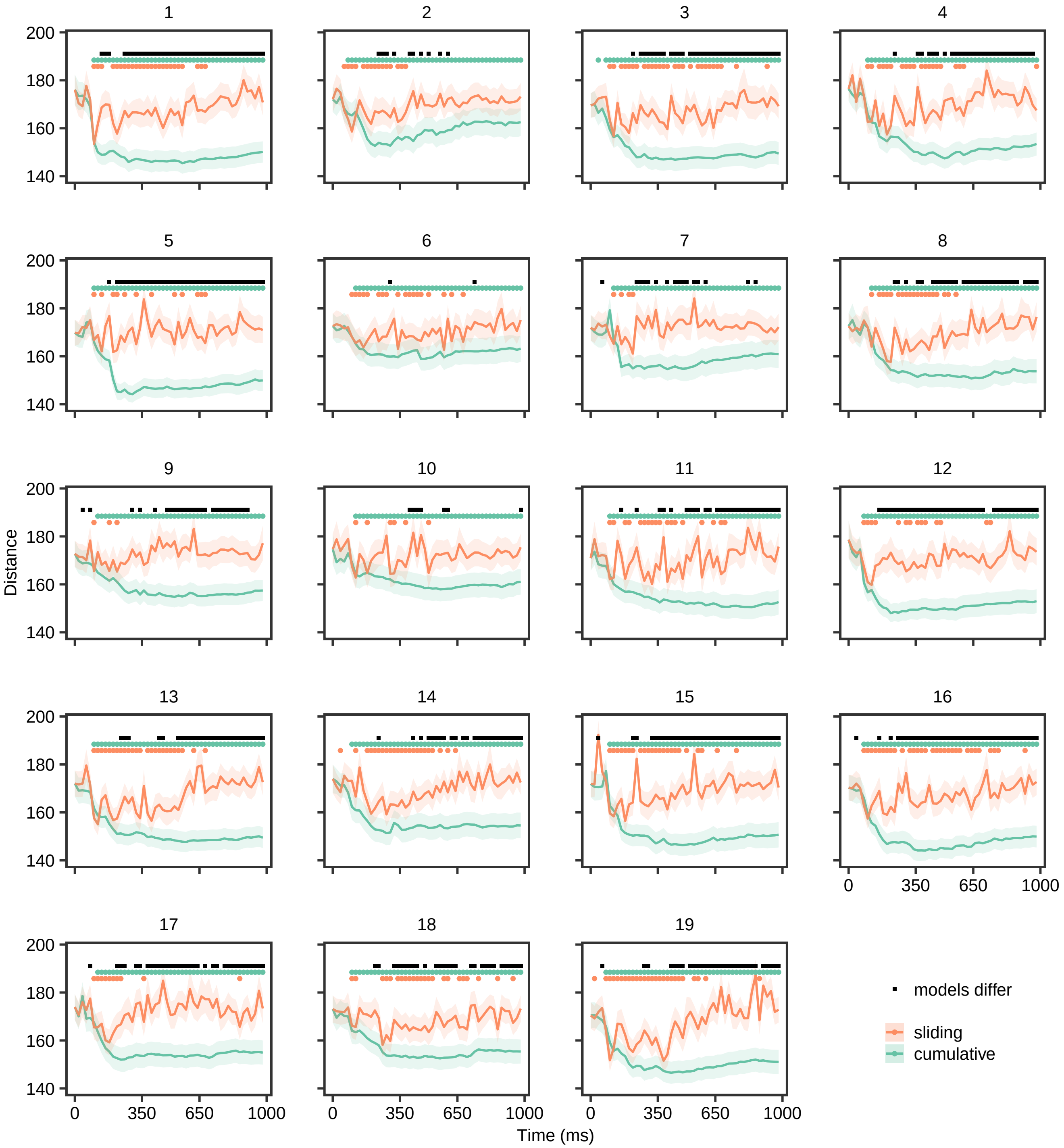


Distance between predictions and targets over time on individual participant data, using two different types of models: a sliding model taking one 20-ms time point at a time, without overlap, and a cumulative window with width increasing at 20-ms increments. The lines represent the mean across concepts, and the shaded areas indicate plus and minus one standard error. The dots above each line plot indicate time points with statistically significant differences ($p < 0.05$, based on permutation tests with 1000 permutations, FDR corrected).

**Supplementary Fig. 2: Distance between predictions and targets over time in individual participants for V2 visual model.**

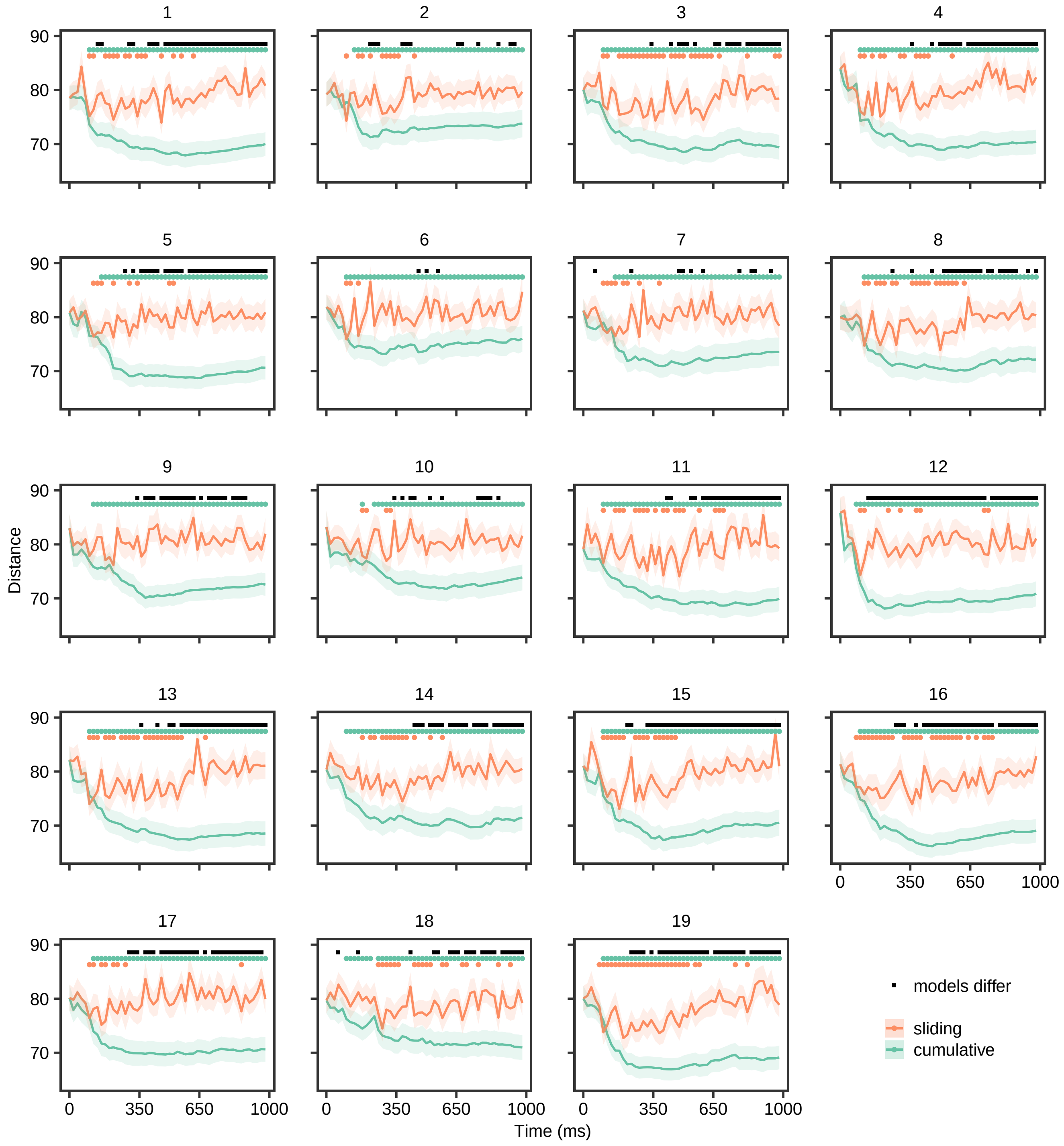


Distance between predictions and targets over time on individual participant data, using two different types of models: a sliding model taking one 20-ms time point at a time, without overlap, and a cumulative window with width increasing at 20-ms increments. The lines represent the mean across concepts, and the shaded areas indicate plus and minus one standard error. The dots above each line plot indicate time points with statistically significant differences ($p < 0.05$, based on permutation tests with 1000 permutations, FDR corrected).

**Supplementary Fig. 3: Distance between predictions and targets over time in individual participants for V4 visual model.**

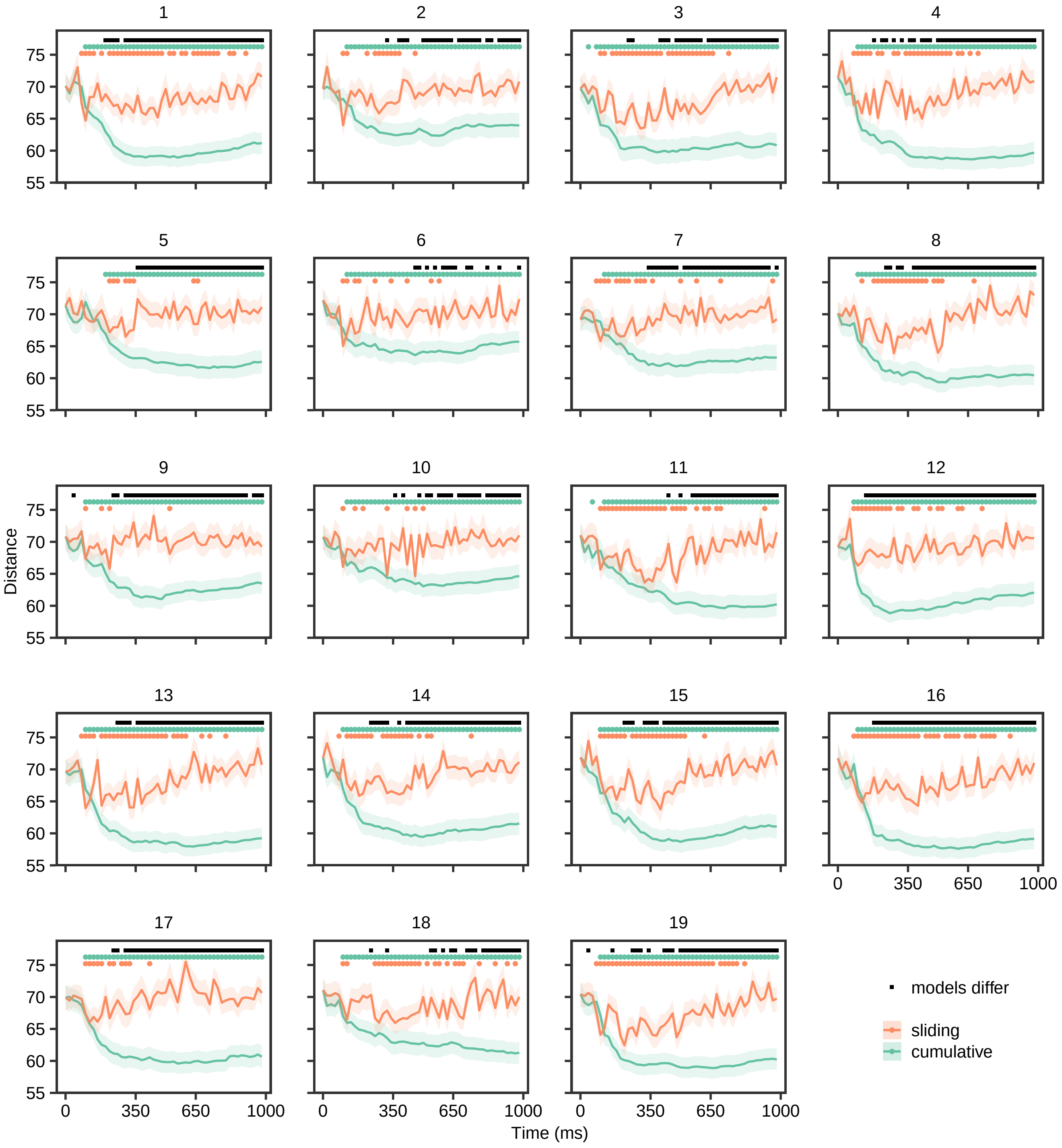


Distance between predictions and targets over time on individual participant data, using two different types of models: a sliding model taking one 20-ms time point at a time, without overlap, and a cumulative window with width increasing at 20-ms increments. The lines represent the mean across concepts, and the shaded areas indicate plus and minus one standard error. The dots above each line plot indicate time points with statistically significant differences ($p < 0.05$, based on permutation tests with 1000 permutations, FDR corrected).

**Supplementary Fig. 4: Distance between predictions and targets over time in individual participants for IT visual model.**

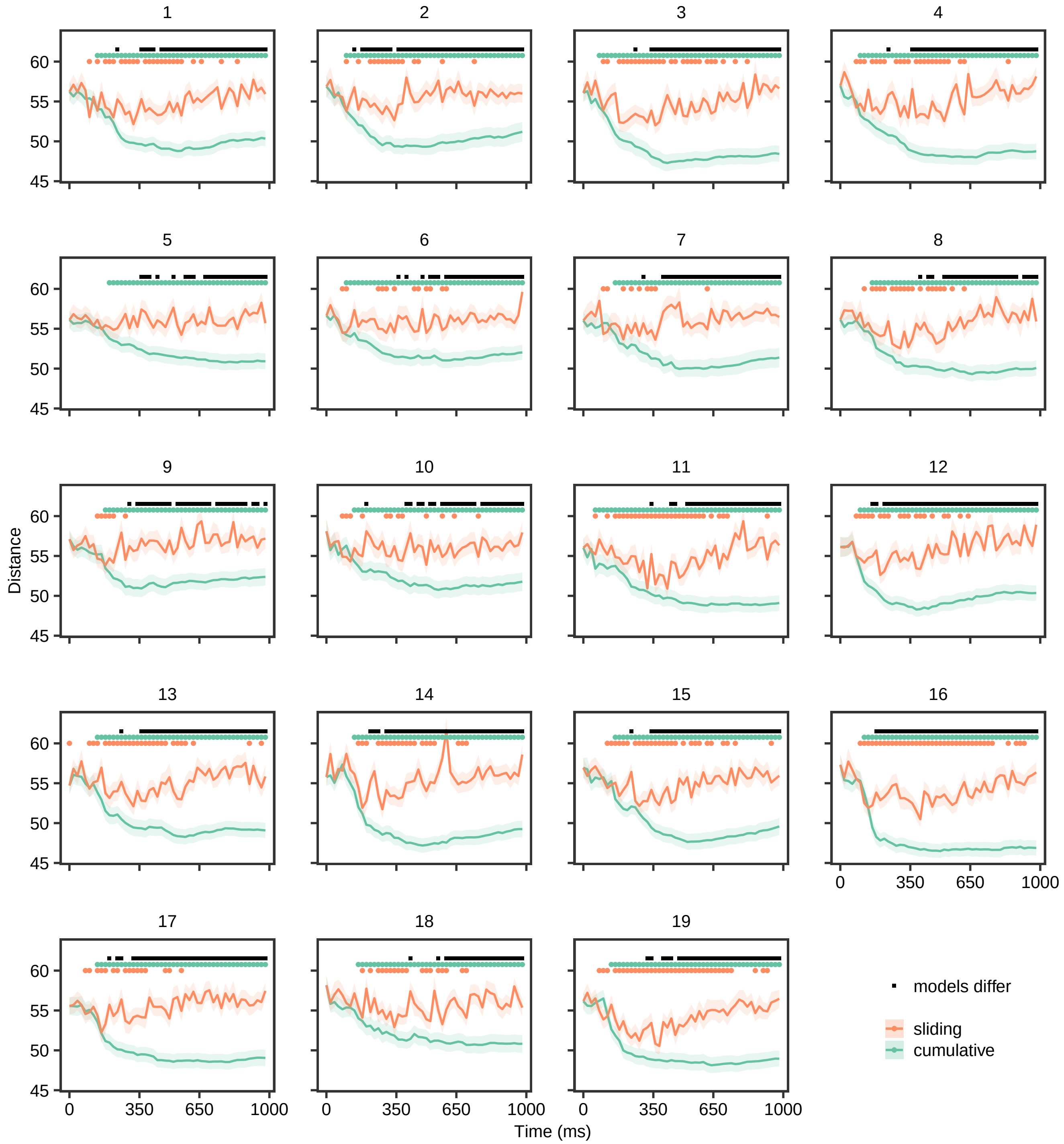


Distance between predictions and targets over time on individual participant data, using two different types of models: a sliding model taking one 20-ms time point at a time, without overlap, and a cumulative window with width increasing at 20-ms increments. The lines represent the mean across concepts, and the shaded areas indicate plus and minus one standard error. The dots above each line plot indicate time points with statistically significant differences ($p < 0.05$, based on permutation tests with 1000 permutations, FDR corrected).

**Supplementary Fig. 5: Correlations between estimated plateau points for different feature models, for each participant.**

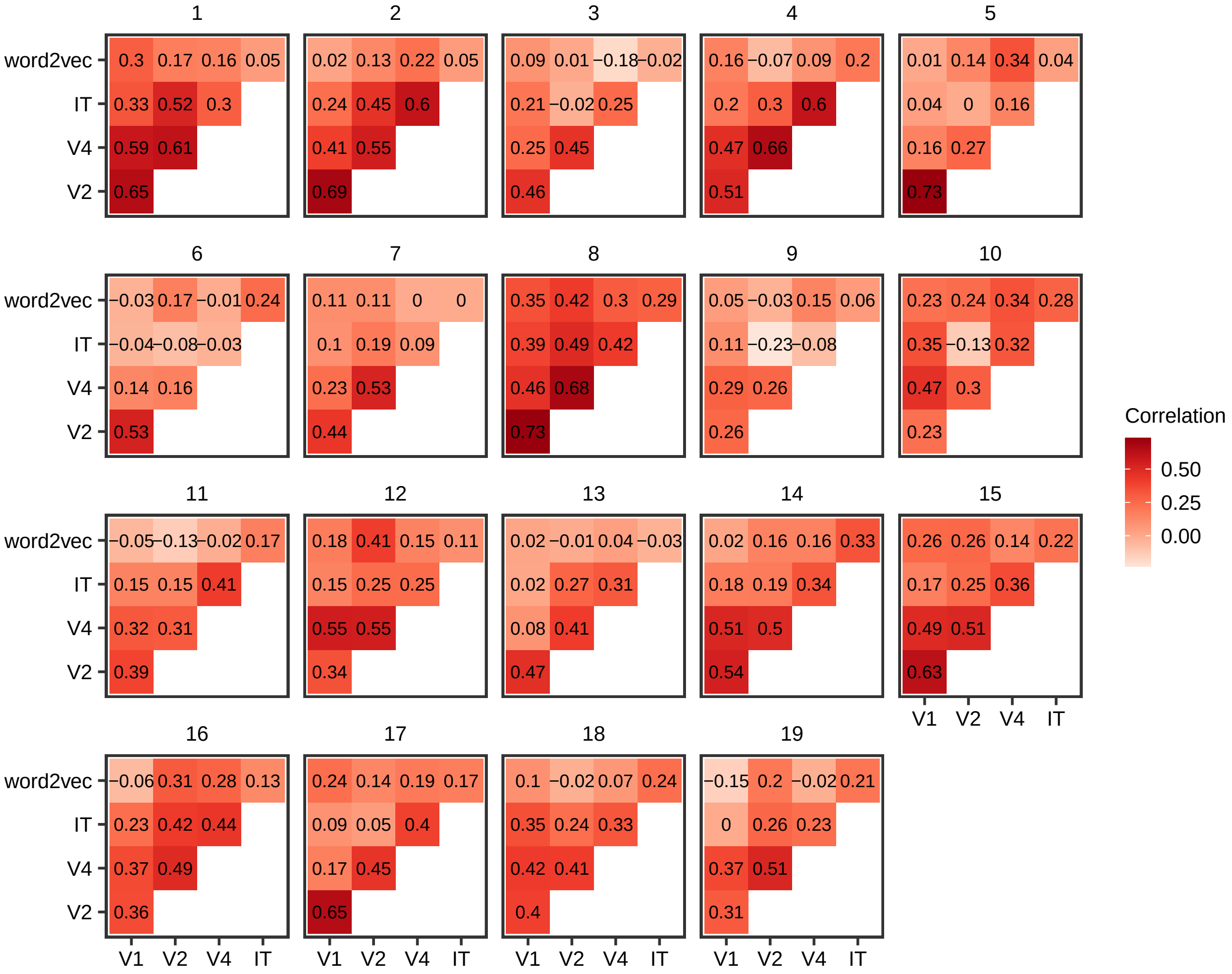

**Supplementary Fig. 6: RSA maps for the visual feature model V1.**

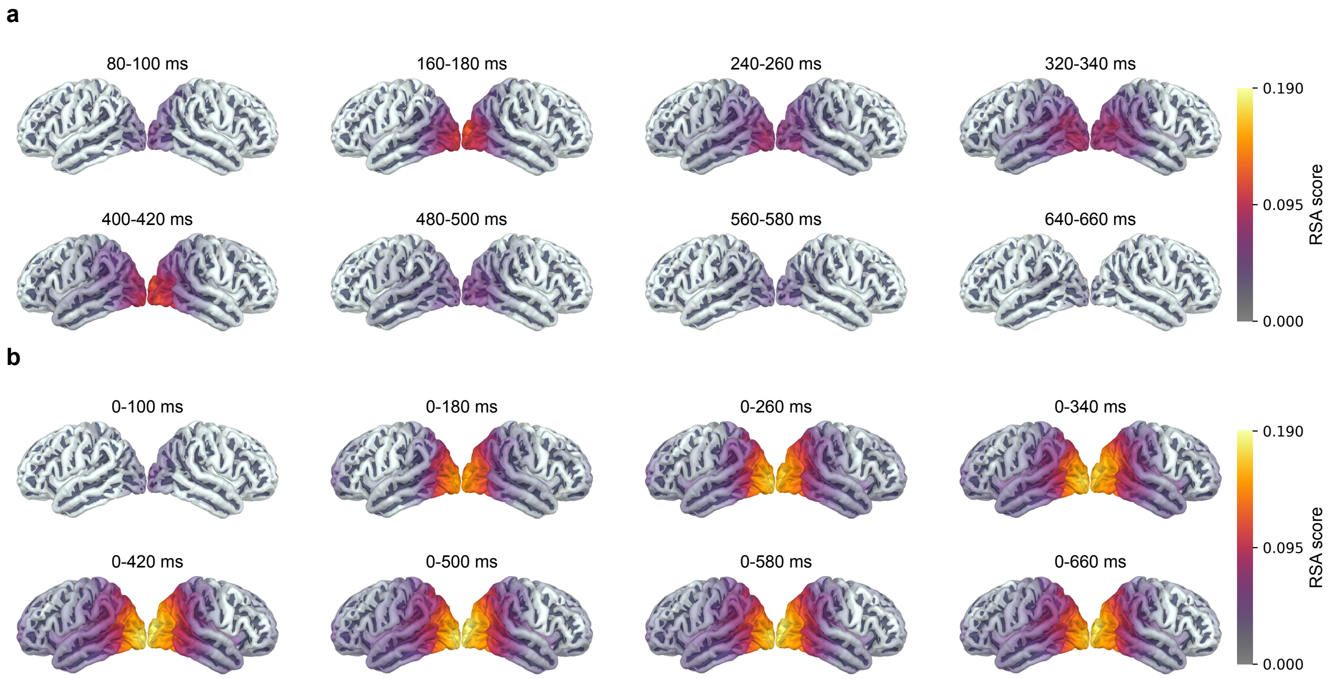


RSA maps illustrating the statistically significant clusters for different time windows, with (**a**) the sliding fixed-length approach and (**b**) the cumulative approach. Significance was determined using a cluster permutation test

**Supplementary Fig. 7: RSA maps for the visual feature model V2.**

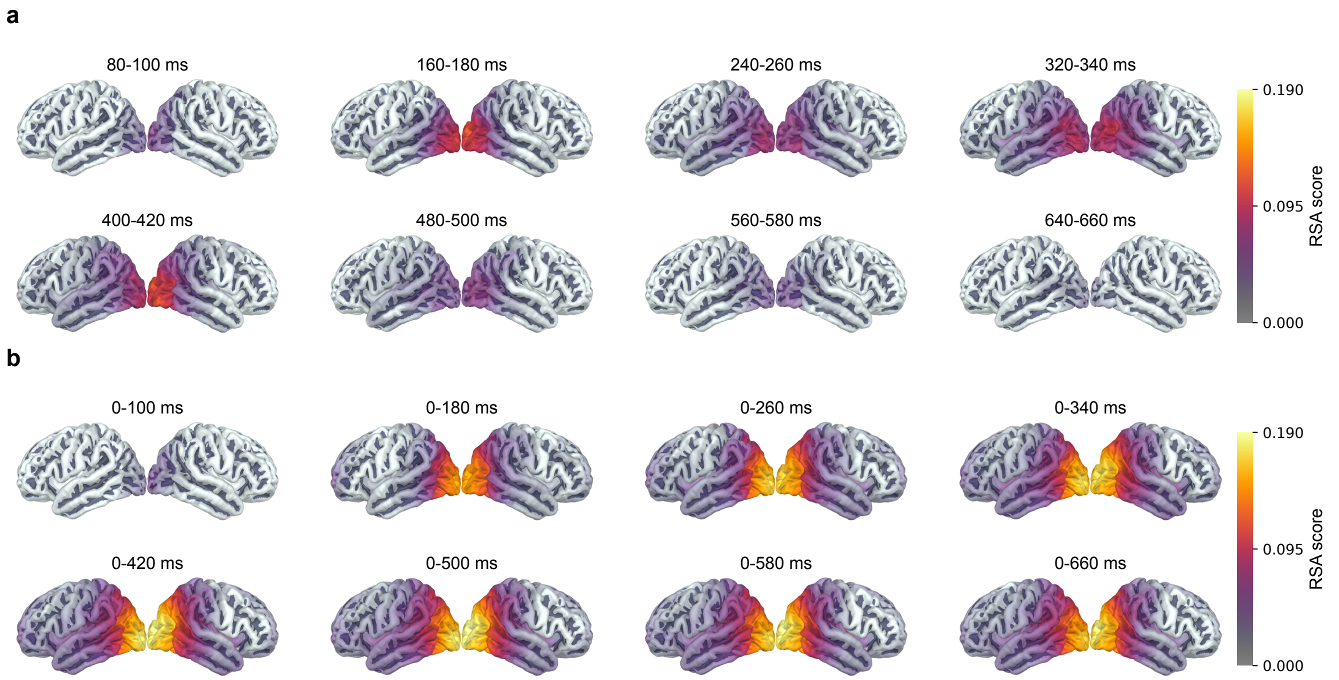


RSA maps illustrating the statistically significant clusters for different time windows, with (**a**) the sliding fixed-length approach and (**b**) the cumulative approach. Significance was determined using a cluster permutation test.

**Supplementary Fig. 8: RSA maps for the visual feature model V4.**

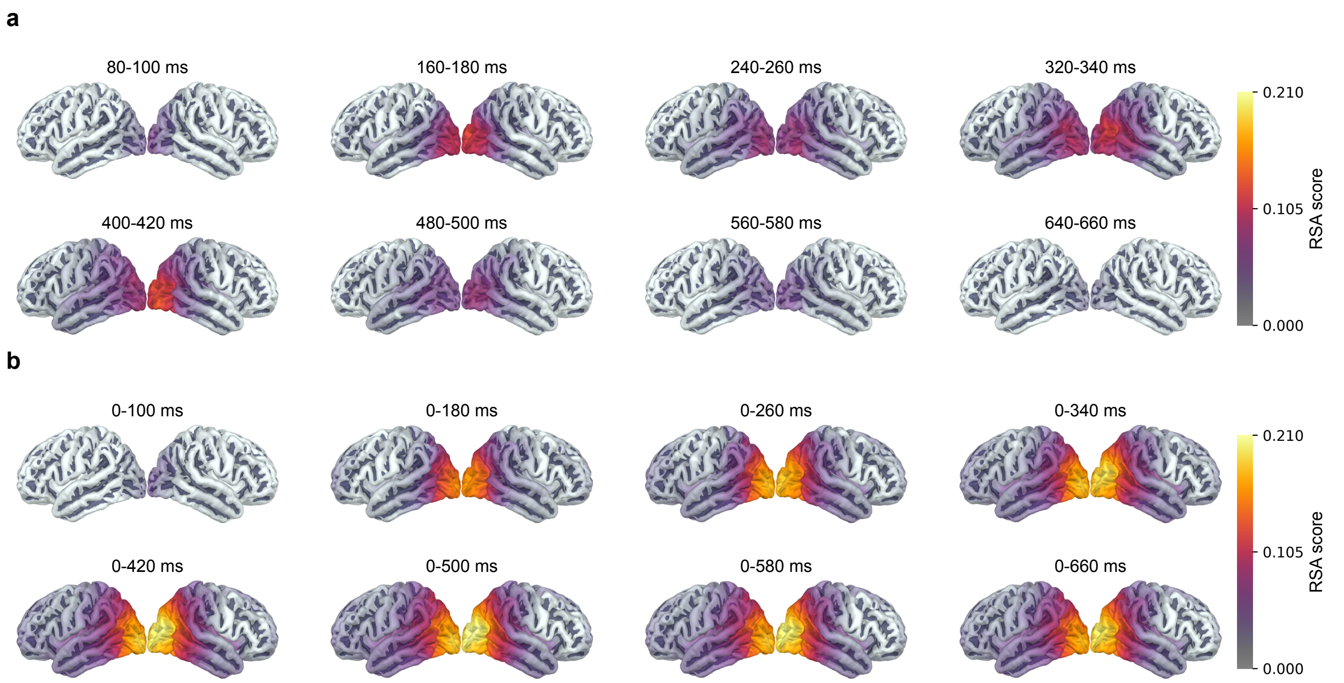


RSA maps illustrating the statistically significant clusters for different time windows, with (**a**) the sliding fixed-length approach and (**b**) the cumulative approach. Significance was determined using a cluster permutation test.

**Supplementary Fig. 9: RSA maps for the visual feature model IT.**

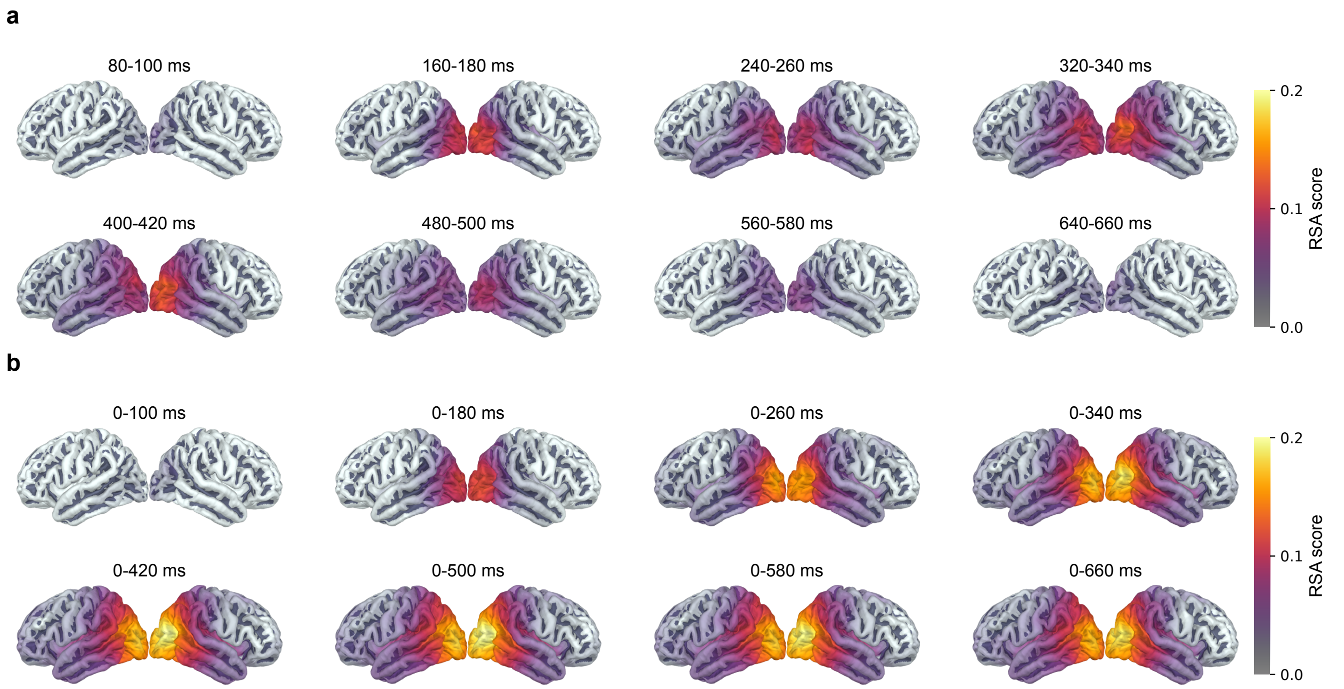


RSA maps illustrating the statistically significant clusters for different time windows, with (**a**) the sliding fixed-length approach and (**b**) the cumulative approach. Significance was determined using a cluster permutation test.
