# Supplementary material for: Cortical time-course of evidence accumulation during semantic processing
Source: Commun Biol. 2023 Dec 8;6:1242. doi: 10.1038/s42003-023-05611-6 (PMC10709650; doi:10.1038/s42003-023-05611-6)
Supplement: Supplementary file 2 — Reporting Summary [file 42003_2023_5611_MOESM2_ESM.pdf]

Corresponding author(s): Gayane Ghazaryan

Last updated by author(s): Nov 13, 2023

Reporting Summary

Nature Portfolio wishes to improve the reproducibility of the work that we publish. This form provides structure for consistency and transparency in reporting. For further information on Nature Portfolio policies, see our Editorial Policies and the Editorial Policy Checklist.

Statistics

For all statistical analyses, confirm that the following items are present in the figure legend, table legend, main text, or Methods section.

n/a	Confirmed	
☐	☒	The exact sample size (*n*) for each experimental group/condition, given as a discrete number and unit of measurement
☐	☒	A statement on whether measurements were taken from distinct samples or whether the same sample was measured repeatedly
☐	☒	The statistical test(s) used AND whether they are one- or two-sided<br>*Only common tests should be described solely by name; describe more complex techniques in the Methods section.*
☒	☐	A description of all covariates tested
☐	☒	A description of any assumptions or corrections, such as tests of normality and adjustment for multiple comparisons
☐	☒	A full description of the statistical parameters including central tendency (e.g. means) or other basic estimates (e.g. regression coefficient) AND variation (e.g. standard deviation) or associated estimates of uncertainty (e.g. confidence intervals)
☐	☒	For null hypothesis testing, the test statistic (e.g. *F*, *t*, *r*) with confidence intervals, effect sizes, degrees of freedom and *P* value noted<br>*Give P values as exact values whenever suitable.*
☒	☐	For Bayesian analysis, information on the choice of priors and Markov chain Monte Carlo settings
☒	☐	For hierarchical and complex designs, identification of the appropriate level for tests and full reporting of outcomes
☐	☒	Estimates of effect sizes (e.g. Cohen's *d*, Pearson's *r*), indicating how they were calculated

Our web collection on statistics for biologists contains articles on many of the points above.

Software and code

Policy information about availability of computer code

Data collection	Presentation Software (https://www.neurobs.com)
Data analysis	MNE Python 1.0.3, scikit-learn 1.1, Freesurfer 7.1, R 3.6, Python 3.9, Maxfilter 2.2, MNE-RSA 0.7dev

For manuscripts utilizing custom algorithms or software that are central to the research but not yet described in published literature, software must be made available to editors and reviewers. We strongly encourage code deposition in a community repository (e.g. GitHub). See the Nature Portfolio guidelines for submitting code & software for further information.

Data

Policy information about availability of data

All manuscripts must include a data availability statement. This statement should provide the following information, where applicable:

- Accession codes, unique identifiers, or web links for publicly available datasets
- A description of any restrictions on data availability
- For clinical datasets or third party data, please ensure that the statement adheres to our policy

The text corpus containing 1.5 billion Finnish words used to derive the statistical model cannot be publicly distributed due to the Finnish copyright law limitations. It is available upon request for research purposes, for contact information see http://bionlp.utu.fi/finnish-internet-parsebank.html. The word2vec models used in this study (derived from the above-mentioned corpus), together with the custom code used in the study can be accessed at https://github.com/AaltoImagingLanguage/ghazaryan2023. The code to compute RSA can be found at https://github.com/wmvanvliet/mne-rsa.

The stimulus words are publicly available and listed in the appendix. The MEG and MRI data are available upon reasonable request from the authors; the data is not publicly available as it contains personal information, and its reuse for other research purposes requires renewed consent from the participants and a new ethical pre-review.

Research involving human participants, their data, or biological material

Policy information about studies with human participants or human data. See also policy information about sex, gender (identity/presentation), and sexual orientation and race, ethnicity and racism.

Reporting on sex and gender	Self-reported gender was collected and reported. Analysis did not directly make use of the gender of the participants, but equal number of males and females participated.
Reporting on race, ethnicity, or other socially relevant groupings	Not recorded.
Population characteristics	See above.
Recruitment	Participants were recruited through university mailing lists. They were volunteers and were compensated for their time.
Ethics oversight	Aalto University Research Ethics Committee

Note that full information on the approval of the study protocol must also be provided in the manuscript.

Field-specific reporting

Please select the one below that is the best fit for your research. If you are not sure, read the appropriate sections before making your selection.

☐ Life sciences ☒ Behavioural & social sciences ☐ Ecological, evolutionary & environmental sciences

For a reference copy of the document with all sections, see nature.com/documents/nr-reporting-summary-flat.pdf

Behavioural & social sciences study design

All studies must disclose on these points even when the disclosure is negative.

Study description	Brain imaging study with quantitative data (MEG measurement).
Research sample	Adult native Finnish speakers, right-handed (aged 20-27 years, mean 22); 10 males, 10 females.
Sampling strategy	Convenience sample. Required sample size was determined based on previous studies, no formal power analysis was performed, but most analysis was done on individual level.
Data collection	Participants viewed stimuli on a computer screen while MEG was recorded. Head position was continuously tracked using coils attached to head. Eye movements were tracked using electrodes. Only research staff and laboratory technicians were present with participants at the laboratory. Researchers were not blinded, but there was no specific experiment manipulation, so it was not deemed necessary.
Timing	June 2015 - August 2015
Data exclusions	Data from one participant was excluded due to technical issues with MEG measurement.
Non-participation	No participants dropped out after beginning participation.
Randomization	Participants were not allocated into experimental groups.

Reporting for specific materials, systems and methods

We require information from authors about some types of materials, experimental systems and methods used in many studies. Here, indicate whether each material, system or method listed is relevant to your study. If you are not sure if a list item applies to your research, read the appropriate section before selecting a response.

Materials & experimental systems

n/a	Involved in the study
☒	☐ Antibodies
☒	☐ Eukaryotic cell lines
☒	☐ Palaeontology and archaeology
☒	☐ Animals and other organisms
☒	☐ Clinical data
☒	☐ Dual use research of concern
☒	☐ Plants

Methods

n/a	Involved in the study
☒	☐ ChIP-seq
☒	☐ Flow cytometry
☐	☒ MRI-based neuroimaging

Magnetic resonance imaging

Experimental design

Design type: Anatomical / structural only images were used to source-localize MEG signals. MRI data was not analyzed directly.

Design specifications: *Specify the number of blocks, trials or experimental units per session and/or subject, and specify the length of each trial or block (if trials are blocked) and interval between trials.*

Behavioral performance measures: *State number and/or type of variables recorded (e.g. correct button press, response time) and what statistics were used to establish that the subjects were performing the task as expected (e.g. mean, range, and/or standard deviation across subjects).*

Acquisition

Imaging type(s): Structural

Field strength: 3 Tesla

Sequence & imaging parameters: *Specify the pulse sequence type (gradient echo, spin echo, etc.), imaging type (EPI, spiral, etc.), field of view, matrix size, slice thickness, orientation and TE/TR/flip angle.*

Area of acquisition: Whole brain scan

Diffusion MRI ☐ Used ☒ Not used

Preprocessing

Preprocessing software: FreeSurfer

Normalization: Intesity normalization: Intensities for all voxels are scaled so that the mean intensity of the white matter is 110.

Normalization template: Talairach

Noise and artifact removal: Automated skull-stripping, then each slice on the aux volume and removed all areas that are separate from the brain.

Volume censoring: *Define your software and/or method and criteria for volume censoring, and state the extent of such censoring.*

Statistical modeling & inference

Model type and settings: *Specify type (mass univariate, multivariate, RSA, predictive, etc.) and describe essential details of the model at the first and second levels (e.g. fixed, random or mixed effects; drift or auto-correlation).*

Effect(s) tested: *Define precise effect in terms of the task or stimulus conditions instead of psychological concepts and indicate whether ANOVA or factorial designs were used.*

Specify type of analysis: ☒ Whole brain ☐ ROI-based ☐ Both

Statistic type for inference: *Specify voxel-wise or cluster-wise and report all relevant parameters for cluster-wise methods.*

(See Eklund et al. 2016)

Correction: *Describe the type of correction and how it is obtained for multiple comparisons (e.g. FWE, FDR, permutation or Monte Carlo).*

Models & analysis

n/a	Involved in the study
☒	☐ Functional and/or effective connectivity
☒	☐ Graph analysis
☒	☐ Multivariate modeling or predictive analysis
